# Implication of mTOR Signaling in NSCLC: Mechanisms and Therapeutic Perspectives

**DOI:** 10.3390/cells12152014

**Published:** 2023-08-07

**Authors:** Antonios N. Gargalionis, Kostas A. Papavassiliou, Athanasios G. Papavassiliou

**Affiliations:** 1 Department of Biopathology, ‘Eginition’ Hospital, Medical School, National and Kapodistrian University of Athens, 11528 Athens, Greece; agargal@med.uoa.gr; 2 First University Department of Respiratory Medicine, ‘Sotiria’ Hospital, Medical School, National and Kapodistrian University of Athens, 11527 Athens, Greece; konpapav@med.uoa.gr; 3 Department of Biological Chemistry, Medical School, National and Kapodistrian University of Athens, 11527 Athens, Greece

**Keywords:** mTOR signaling, non-small cell lung cancer (NSCLC), PI3K, Akt, rapamycin

## Abstract

Mechanistic target of the rapamycin (mTOR) signaling pathway represents a central cellular kinase that controls cell survival and metabolism. Increased mTOR activation, along with upregulation of respective upstream and downstream signaling components, have been established as oncogenic features in cancer cells in various tumor types. Nevertheless, mTOR pathway therapeutic targeting has been proven to be quite challenging in various clinical settings. Non-small cell lung cancer (NSCLC) is a frequent type of solid tumor in both genders, where aberrant regulation of the mTOR pathway contributes to the development of oncogenesis, apoptosis resistance, angiogenesis, cancer progression, and metastasis. In this context, the outcome of mTOR pathway targeting in clinical trials still demonstrates unsatisfactory results. Herewith, we discuss recent findings regarding the mechanisms and therapeutic targeting of mTOR signaling networks in NSCLC, as well as future perspectives for the efficient application of treatments against mTOR and related protein molecules.

## 1. Introduction

Lung cancer is the second leading cause of cancer incidence and the first leading cause of cancer deaths globally [1]. Non-small cell lung cancer (NSCLC) affects around 80 to 85% of lung cancer patients and is divided into 3 main types: adenocarcinoma, large cell carcinoma, and squamous cell carcinoma [2]. NSCLC features various molecular alterations which drive oncogenesis and are implicated in therapeutic targeting and efficacy. These alterations include *epidermal growth factor receptor* (*EGFR*) mutations, *anaplastic lymphoma kinase* (*ALK*) and *c-ros oncogene 1* (*ROS1*) rearrangements, as well as *v-Raf murine sarcoma viral oncogene homolog B* (*BRAF*), *mesenchymal-epithelial transition factor* (*MET*), *rearranged during transfection* (*RET*), *neurotrophic tyrosine receptor kinase* (*NTRK*) genetic alterations [3]. Classic treatment options are surgical resection, adjuvant, and neoadjuvant chemotherapy. Based on extensive molecular profiling of NSCLC, several types of targeted treatment have been developed with the application of EGFR inhibitors and monoclonal antibodies, other Tyr kinase inhibitors, vascular endothelial growth factor receptor (VEGFR) inhibitors, as well as immunotherapy either as monotherapy or in combination regimens [2].

The mTOR signaling cassette has been intensely implicated in NSCLC pathobiology. Genetic alterations of respective genes have been identified in subsets of NSCLC patients. Furthermore, the upregulation of mTOR itself, of upstream signaling molecules and downstream substrates has been documented in large NSCLC cohorts and it has been correlated with other known activating mutations. However, clinical trials of mTOR pathway-targeting compounds provided unsatisfactory results so far [4]. Herein, we discuss the implication of the mTOR pathway in NSCLC and current concerns regarding the efficient application of mTOR pathway inhibitors in NSCLC clinical settings.

## 2. Structure and Functions of the mTOR Signaling Pathway

**Figure 1:**
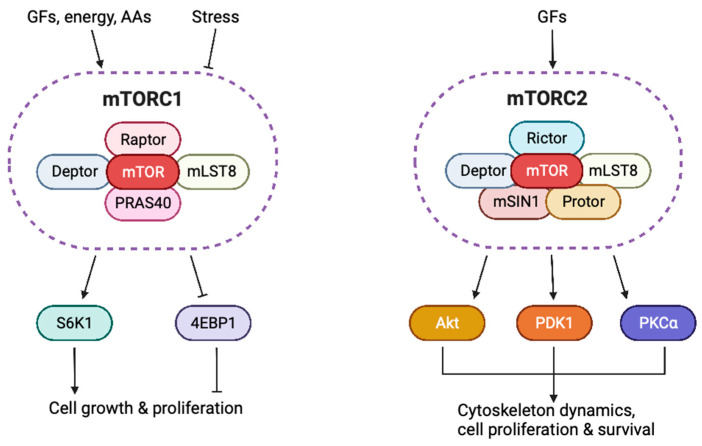
Schematic representation of both mTOR complexes, mTORC1 and mTORC2, along with their upstream activating stimuli and downstream effectors. GFs, growth factors; AAs, amino acids; mTORC1, mTOR complex 1; mTORC2, mTOR complex 2; mLST8, mammalian lethal with SEC13 protein 8; Deptor, DEP-domain-containing-mTOR-interacting protein; Raptor, regulatory-associated protein of mTOR; PRAS40, proline-rich AKT substrate 40 kDa; mSIN1, mitogen-activated protein kinase (MAPK)-interacting protein 1; Protor, protein associated with rictor; Rictor, rapamycin-insensitive companion of mammalian target of rapamycin; S6K1, p70 S6 kinase 1; 4EBP1, 4E-binding protein 1; Akt, protein kinase B; PDK1, phosphoinositide-dependent kinase-1; PKCα, protein kinase Cα. This figure was created based on the tools provided by Biorender.com (accessed on 27 July 2023).

The mTOR kinase has been identified as the target of the macrolide termed rapamycin. Rapamycin has been attributed to antifungal, immunosuppressive, antitumor, and neuroprotective activities [5]. mTOR is a Ser/Thr protein kinase and a member of the phosphoinositide 3-kinase (PI3K)-related protein kinases (PIKK) family. There are two complexes that share mTOR as a catalytic subunit: mTOR complex 1 (mTORC1) and mTOR complex 2 (mTORC2) (Figure 1). This mTOR-shared domain consists of clusters of huntingtin, elongation factor 3, a subunit of protein phosphatase 2A and TOR1 (HEAT) repeats at the amino-terminal end, the FRAP, ATM, and TRRAP (FAT) domain, the FKBP12–rapamycin binding (FRB) domain, the catalytic kinase domain, and the carboxy-terminal FATC domain [6].

The mTORC1 bears the mTOR-shared domain, along with the following structures: mammalian lethal with SEC13 protein 8 (mLST8; also known as G*β*L), the inhibitor of mTORC1 activity DEP-domain-containing-mTOR-interacting protein (DEPTOR), and regulatory-associated protein of mTOR (RAPTOR), which is an evolutionarily conserved subunit that defines mTORC1, upregulates nutrients-induced signaling, and downregulates mTOR kinase activity [7]. RAPTOR associates with an additional endogenous inhibitor, proline-rich AKT substrate 40 kDa (PRAS40) [5,6,8,9]. The mTORC1 is inhibited by rapamycin and integrates information from the microenvironment regarding O_2_, energy and nutrient supply, growth factors, and stress. Thereby, mTORC1 suppresses autophagy and facilitates nucleotide, lipid, and protein synthesis to promote cellular growth [5]. The mTORC1 phosphorylates downstream protein substrates such as p70 S6 kinase 1 (S6K1), eukaryotic initiation factor 4E-binding proteins (EIF4E-BPs), and S6 ribosomal protein (S6rp) and modulates the activity of transcription factors, such as lipid-related sterol regulatory element-binding protein 1/2 (SREBP1/2), stress-related activating transcription factor 4 (ATF4), peroxisome proliferator-activated receptor-γ (PPARγ), PPARγ coactivator 1α (PGC1α), energy-related hypoxia-inducible factor 1α (HIF1α), autophagy-related transcription factor EB (TFEB), and mitochondria-related yin–yang 1 (YY1) [6,10,11,12]. The mTORC1 upstream regulators promote mTORC1 activation when nutrients and energy are available and include the small GTPase Rheb in its GTP-bound state, Rag guanosine-5′-triphosphate (GTP)ases, and tuberous sclerosis complex (TSC) as a GTPase-activating protein (GAP) for Rheb [13,14] (Figure 1).

mTORC2 comprises mLST8, the RICTOR scaffolding protein, mitogen-activated protein kinase (MAPK)-interacting protein 1 (mSIN1), DEPTOR, and protein associated with rictor 1 or 2 (PROTOR1/2) [15,16]. mTORC2 mediates cytoskeletal rearrangements and fosters mechanisms of proliferation and survival [5]. The mTORC2 phosphorylates Akt through phosphoinositide-dependent kinase-1 (PDK1) [17]. The mTORC2 downstream protein substrates include Akt, protein kinase Cα (PKCα), and serum- and glucocorticoid-induced protein kinase 1 (SGK1). The mTORC2 interacts with mTORC1 through Akt, which suppresses glycogen synthase kinase 3*β* (GSK3*β*) and tuberous sclerosis complex 2 (TSC2), to finally repress mTORC1 [6]. The mTORC2 activity is regulated by growth factors via the PI3K pathway [18,19] (Figure 1).

## 3. mTOR Signaling in Cancer

**Figure 2:**
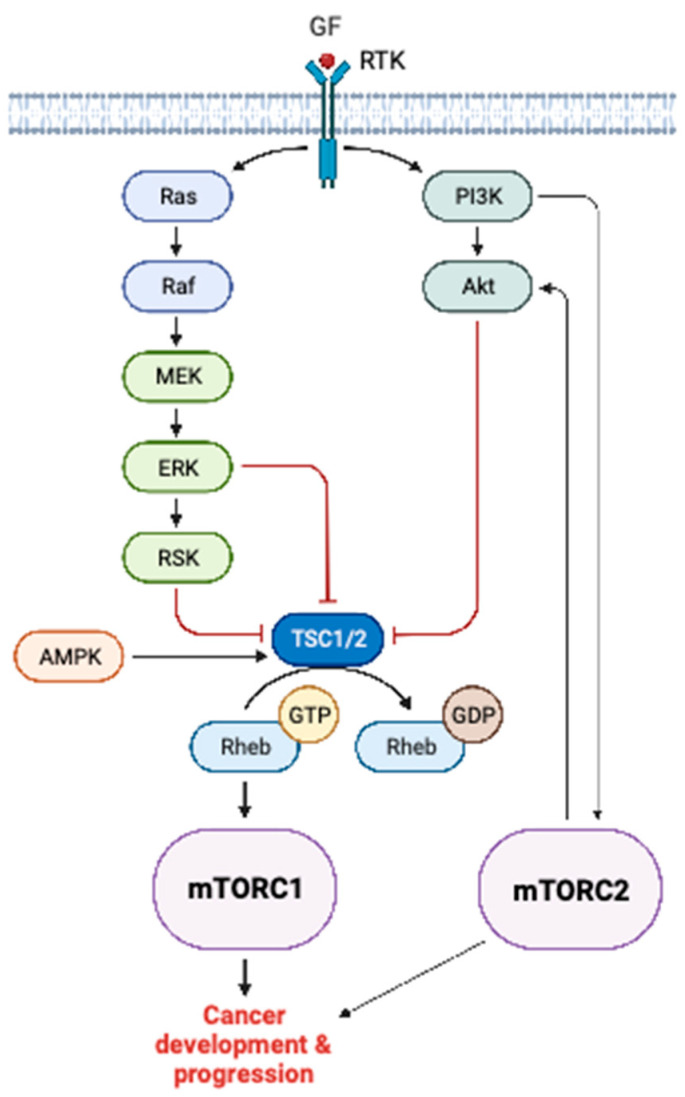
Illustration of mTOR signaling in cancer. mTORC1 is activated by receptor tyrosine kinase signaling mediated through PI3K/Akt signaling and/or Ras/Raf/MEK/ERK signaling. mTORC2 activates mTORC1 via Akt potentiation. GF, growth factor; RTK, receptor tyrosine kinase; PI3K, phosphoinositide 3-kinase; Akt, protein kinase B; Ras, rat sarcoma virus; Raf, rapidly accelerated fibrosarcoma; MEK, mitogen-activated protein kinase kinase; ERK, extracellular signal-related kinase; RSK, 90 kDa ribosomal S6 kinase; AMPK, AMP-activated protein kinase; TSC1/2, tuberous sclerosis complex 1 and 2; Rheb, ras homolog enriched in brain; GTP, guanosine-5′-triphosphate; GDP, guanosine diphosphate. This figure was created based on the tools provided by Biorender.com (accessed on 27 July 2023).

The mTOR is a central hub for several oncogenic signaling pathways in cancer cells and therefore it is activated in a plethora of human malignancies [5]. Data based on solid tumors suggest that mTOR signaling is dysregulated in approximately 30% of cancers, making it one of the most frequently affected signaling pathways in human cancers [20]. For example, the PI3K/Akt/mTOR pathway is deregulated in the majority (90%) of lung adenocarcinomas and 40% of squamous cell lung carcinomas [21]. Other human cancers associated with aberrant mTOR signaling include breast cancer, prostate cancer, colorectal cancer, and gliomas [22,23,24,25]. Mechanistically, mTOR integrates upstream signals when PI3K is activated by receptor tyrosine kinases (RTKs), oncoproteins of the RAS family, Akt kinase, and phosphatase and tensin homolog (PTEN) loss-of-function [5,26,27]. Upstream mTOR activation includes cancer-associated genetic alterations of the *phosphatidylinositol-4,5-bisphosphate 3-kinase*, *catalytic subunit alpha* (*PIK3CA*), *mTOR*, *Akt*, and *PTEN* genes [28]. Specifically, mTORC1 is activated when TSC1/2 is suppressed either from Akt or from mitogen-activated protein kinases (MAPKs) family members. Inactivation of TSC1/2 results in the conversion of Rheb to its GTP-bound state, which, in turn, leads to mTORC1 activation [5]. The mTOR presents a variety of downstream effectors through which oncogenic activating mechanisms impair normal cellular processes. The mTORC1 functions through S6K1, 4E-BP1, EIF4E, and S6rp to impede protein translation. mTORC1 dysregulation affects the downstream activity state of SREBPs, ATF4, and HIF1α, thus leading to abnormal lipogenesis, stress responses, nucleotide synthesis, and aerobic glycolysis. mTORC2 can potentiate Akt and, hence, activate mTORC1 (Figure 2). The mTORC2 also regulates cytoskeletal rearrangements through protein kinase C (PKC), glucose homeostasis through GSK3*β* and cell cycle progression, proliferation, and apoptosis through Ser/Thr protein kinase SGK/forkhead box O1/3a (FOXO1/3a) axis [5].

Dysregulated mTOR signaling promotes survival and cell proliferation, while it suppresses apoptosis. The mTOR also favors angiogenesis, stress responses and DNA damage, cell migration, metastasis, and epithelial-to-mesenchymal transition (EMT) [28,29]. Activation of the PI3K–Akt–mTOR axis triggers autophagy and tumor cells become able to adapt to the environmental status of low nutrients and, therefore, survive. The mTOR also mediates resistance to various chemotherapeutic regimens [28]. Furthermore, mTOR emerges as a critical regulator of the tumor microenvironment (TME) and immune responses. The mTOR signaling molecules participate in differentiation as well as in processes of both innate and adaptive immunity. The mTOR controls regulatory T cells (TREG)/T helper 17 (Th17) homeostasis in the TME and can facilitate polarization of tumor-associated macrophage into M2 macrophage. It also drives the accumulation of tumor-induced myeloid-derived suppressor cells to stimulate tumor progression, and mTOR-dependent mechanisms in TMEs polarize neutrophils toward pro-tumoral phenotypes. Moreover, mTOR mediates modulation of dendritic cells in the TME, but also regulates natural killer (NK) cells and their proliferation and metabolic activity [30].

## 4. mTOR Signaling in NSCLC Pathobiology

Several oncogenic alterations have been reported regarding components of the mTOR signaling axis in the context of lung cancer. As far as upstream molecules of mTOR signaling are concerned, alterations affecting PIK3CA, Akt, and PTEN are the most common and important with respect to clinicopathological features. The *PIK3CA* gene may be altered in NSCLC by way of constitutively-activating mutations or via increasing the gene copy number [31]. These genetic aberrations have been associated with gender, smoking status, NSCLC subtype, disease stage, lymph node metastasis, progression-free survival, and overall survival [32,33,34,35,36]. Similarly, *Akt* gene alterations in the setting of lung cancer include activating mutations or gene overexpression and are linked to advanced disease stage, lymph node metastasis, and poor survival [37,38,39,40]. As a tumor suppressor gene, *PTEN* undergoes mutations that lead to decreased or loss of expression of PTEN protein [41,42,43]. A significant proportion of NSCLCs presents upregulated mTOR signaling. The activated form of mTOR, phosphorylated(p)-mTOR, has been found to be increased in the majority (~90%) of adenocarcinoma patients, ~60% of large cell carcinoma patients, and ~40% of squamous cell carcinoma patients [44,45,46]. 4E-BP1 and S6K1 (the downstream effectors of activated mTOR), also displayed augmented expression in NSCLC biopsies, particularly those derived from lung adenocarcinomas [21]. Importantly, increased protein expression of mTOR was correlated with poor survival in early-stage NSCLC [47,48].

Deregulation of the mTOR pathway promotes tumorigenesis and malignant progression in NSCLC. Specifically, aberrant mTOR signaling participates in the acquisition of multiple important hallmarks of cancer cells such as uncontrolled cell proliferation, inhibition of apoptosis and autophagy, angiogenesis, migration and invasion, and metastasis. For example, several studies showed that Akt inhibition induced apoptosis in A549 lung adenocarcinoma cells [49,50,51,52]. In addition, inhibition of mTOR signaling was demonstrated to induce autophagy in lung adenocarcinoma [53,54,55]. Moreover, studies emphasize the role of PI3K signaling and PTEN in the promotion of migration, invasion and metastasis in lung adenocarcinoma [56,57,58]. Furthermore, there is enough evidence supporting the role of the mTOR pathway in angiogenesis in lung adenocarcinoma via affecting the expression of VEGF [59,60,61,62]. The mTOR signaling also exerts a key role in mediating resistance to chemotherapeutic drugs, radiotherapy, targeted therapy, and immunotherapy in NSCLC patients [4,48,63,64,65]. Particularly, the loss of PTEN and PIK3CA mutations have been strongly associated with resistance to EGFR inhibitors in lung adenocarcinoma [66,67,68,69,70].

## 5. Therapeutic Targeting of mTOR Signaling in NSCLC: Current Status

**Figure 3:**
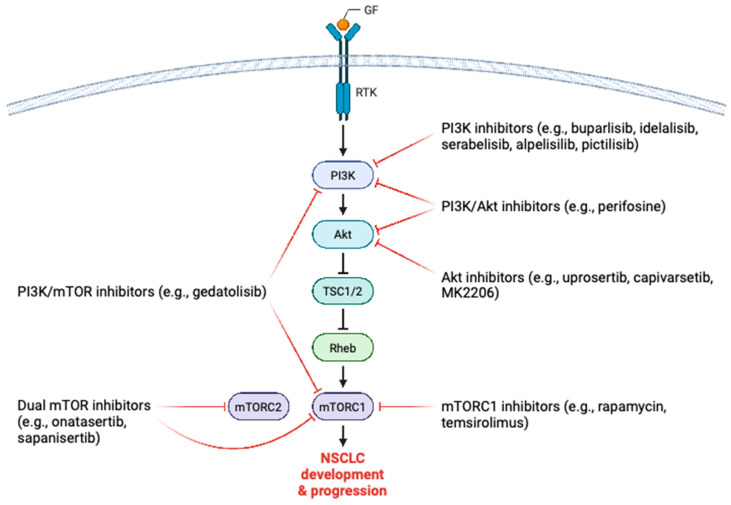
Illustration of pharmacological modulation of the mTOR pathway in the setting of NSCLC. Various small molecule inhibitors have been developed and are under clinical investigation targeting different mTOR signaling components. GF, growth factor; RTK, receptor tyrosine kinase; PI3K, phosphoinositide 3-kinase; Akt, protein kinase B; TSC1/2, tuberous sclerosis complex 1 and 2; Rheb, ras homolog enriched in brain; NSCLC, non-small cell lung cancer. This figure was created based on the tools provided by Biorender.com (accessed on 27 July 2023).

Given the involvement of the mTOR signaling pathway in the molecular mechanisms underpinning NSCLC pathobiology, it is no wonder that preclinical and clinical research has focused on the pharmacological targeting of this important oncogenic signaling pathway for the potential benefit of patients with NSCLC. Pharmacological modulation of the mTOR axis includes targeting upstream activating molecules, such as PI3K and Akt, as well as direct targeting of the mTOR complexes (Figure 3).

Several PI3K-targeting drugs have been developed, including selective PI3K inhibitors, pan-PI3K inhibitors, and dual PI3K/mTOR or PI3K/Akt inhibitors. Buparlisib (BKM120) is a class I pan-PI3K inhibitor that is presently being evaluated in clinical trials in combination with the EGFR inhibitor gefitinib, the chemotherapeutics cisplatin and etoposide, and carboplatin and pemetrexed (NCT01570296—phase I, NCT02194049—phase I, NCT01723800—phase I). Idelalisib, a PI3Kδ inhibitor, combined with the immune checkpoint inhibitor pembrolizumab is tested in an ongoing clinical trial involving NSCLC patients unresponsive to immunotherapy (NCT03257722—phase I/II). Serabelisib, which inhibits PI3Kα, is under clinical investigation as a treatment for advanced NSCLC tumors in combination with canagliflozin (NCT04073680—phase I/II). Similarly, alpelisib, targeting PI3Kα, in combination with the MEK inhibitor MEK162, is under assessment in a clinical trial including advanced NSCLC patients (NCT01449058—phase I). Pictilisib is a PI3Kα/δ inhibitor that is currently undergoing clinical evaluation in advanced NSCLC in combination with many different chemotherapeutic regimens (NCT00974584—phase I). In the class of dual PI3K/mTOR inhibitors, gedatolisib is being investigated in separate trials in combination with chemotherapies and the cyclin-dependent kinase 4/6 (CDK4/6) inhibitor palbociclib (NCT03065062—phase I). Uprosertib, capivarsetib, and MK2206 are all small-molecule Akt inhibitors and clinical trials are currently underway to assess their efficacy in combination with other agents in NSCLC patients (NCT01147211—phase I). Perifosine, belonging to the class of dual PI3K/Akt inhibitors, is in the process of clinical investigation in NSCLC patients. Treatment with mTORC1 inhibitors rapamycin or temsirolimus and other therapies, such as chemotherapy, targeted therapy, and radiotherapy, is presently being evaluated in clinical trials conducted in NSCLC patients (NCT00555256—phase I, NCT00796796—phase I). Finally, the dual mTOR inhibitors onatasertib and sapanisertib are undergoing clinical trials for the treatment of advanced NSCLC patients (NCT01545947—phase I, NCT04250545—phase II).

## 6. Outlook and Future Perspectives

Recent studies are developing new mTOR inhibitors with better selectivity and more potent anti-cancer effects on NSCLC cells [71]. There are also novel pharmacological agents with dual blocking action capable of modulating two different targets at once. For example, CC-115 represents a novel drug that can potently inhibit both mTORC1 and mTORC2, as well as DNA-dependent protein kinase (DNA-PK) and has demonstrated suppression of NSCLC growth both in vivo and in vitro [72]. Interestingly, research is also revealing new effects of mTOR inhibition on cancer cells which can be exploited for further therapeutic targeting [73]. A recent study suggests the combinatorial use of mTOR pathway inhibitors and immune checkpoint inhibitors as oncogenic mTOR signaling can regulate the expression of programmed death ligand 1 (PD-L1) in NSCLC [42]. Nevertheless, as pointed up for other solid tumor types, e.g., breast cancer with highly dubious microcalcifications [74,75], stage-specific expression/activation profile of relevant signaling molecules and immunological status should also be taken into consideration in formulating targeted combinatorial therapies for NSCLC. The terminal effects of mTOR signaling in NSCLC cells are mediated via the action of transcription factors, ultimately leading to gene expression reprogramming. Thus, combined targeting of mTOR pathway components and transcription factors/cofactors may be a promising therapeutic strategy in the clinical management of NSCLC patients [76].

The plethora of different molecular profiles of NSCLCs combined with the complex interplay of the mTOR pathway with other signaling pathways cannot be overlooked during the process of drug development, since these biological aspects will most probably hinder the successful application of mTOR pathway-targeting drugs in the oncology clinic. Therefore, research in this area should consider therapeutic strategies that take into account the molecular profile of NSCLC tumors with respect to mTOR signaling and employ combination therapies. Identifying predictive and prognostic biomarkers associated with mTOR signaling will be useful in guiding the clinical management of NSCLC patients whose tumors exhibit aberrations in this oncogenic pathway [77].

## 7. Conclusions

NSCLC is a highly heterogeneous disease that can be driven by a variety of distinct genetic alterations in lung epithelial cells, resulting in vastly different tumor phenotypes in each patient. The mTOR pathway undergoes a plethora of genetic alterations in the context of NSCLC which contribute to tumorigenesis and malignant progression. Several studies have revealed the crucial role of aberrant mTOR signaling in the promotion of cancer cell hallmarks, such as uncontrolled proliferation, apoptosis inhibition, migration and invasion, metastasis, angiogenesis, and resistance to drugs. Additional studies imply a strong correlation between the expression status of mTOR pathway components and clinical prognosis. Furthermore, preclinical in vivo and in vitro research on pharmacological modulation of the mTOR pathway in NSCLC models has provided promising results. Lastly, many ongoing clinical trials evaluate different small molecule inhibitors targeting components of the mTOR cascade alone or in combination with other agents whose results are eagerly awaited.

## Data Availability

No new data were created.
